# Effectiveness of the Intervention from a Mental Health Day Hospital with Patients with Various Mental Disorders in Burgos, Spain

**DOI:** 10.3390/jcm14186538

**Published:** 2025-09-17

**Authors:** Sandra Núñez-Rodríguez, Patricia Olano-Isasi, Manuel Mateos-Agut, Xosé Ramón García-Soto, Beatriz Sanz-Cid, Álvaro García-Bustillo, Jerónimo J. González-Bernal, Josefa González-Santos

**Affiliations:** 1 Department of Health Sciences, University of Burgos, 09001 Burgos, Spain; snunez@ubu.es (S.N.-R.); poi0002@alu.ubu.es (P.O.-I.); mjgonzalez@ubu.es (J.G.-S.); 2 Psychiatry Service, University Hospital of Burgos, 09006 Burgos, Spain; mmateosa@saludcastillayleon.es (M.M.-A.); xgarcia@saludcastillayleon.es (X.R.G.-S.); bsanz09@hotmail.com (B.S.-C.)

**Keywords:** mental health, mental disorders, psychiatry, psychiatric hospital, psychiatric intervention, Spain

## Abstract

**Background/Objectives**: Mental health disorders represent a growing challenge for healthcare systems worldwide. The day hospital model has established itself as an effective strategy for outpatient treatment. This study aims to evaluate the effectiveness of the Mental Health Day Hospital at University Hospital of Burgos, as well as to analyze the influence of sociodemographic factors on the clinical evolution of patients. **Methods**: This was a retrospective, single-center study with repeated cross-sectional assessments with pre- and post-tests, analyzing data from 1629 patients over the age of 18 treated between 1996 and 2022 at the Mental Health Day Hospital of the University Hospital of Burgos, in Spain, of whom 653 completed the pre-test and post-test. **Results**: Differences in prevalence were observed by sex and age, with a higher frequency of eating disorders in women (92.5%) and substance use disorders in men (67.9%). The average age varied according to diagnosis, being highest in mood disorders (43.00) and lowest in eating disorders (23.00). Significant correlations were observed between most variables (*p* < 0.05), especially between anxiety, impulsiveness, and self-esteem symptoms. The overall reduction in symptoms validates the program′s effectiveness, although less improvement was identified in self-esteem and assertiveness, especially in psychotic disorders. Furthermore, patients with anxiety disorders showed a lower response in trait anxiety. **Conclusions**: The study highlights the importance of tailoring interventions according to each patient′s sex, age, and diagnosis. Optimizing treatments based on these variables will improve care and therapeutic outcomes, especially for those with more complex disorders.

## 1. Introduction

Mental health disorders represent a growing challenge for public health systems globally, affecting millions of people in terms of quality of life, work productivity and general well-being [1,2,3]. According to recent data from the World Health Organization (WHO), approximately 1 in 8 people worldwide, or 970 million individuals, suffer from a mental disorder. Among the most common are anxiety disorders, which affect more than 301 million people, and depressive disorders, with a prevalence of more than 280 million cases worldwide [4]. In the national context, the most recent primary care surveys indicate that the prevalence of mental health disorders is 27.4%. In Spain, the most common are anxiety disorders, which affect 6.7% of the Spanish population, followed by depressive disorders, which affect 4.1% of the population. Schizophrenia and other psychotic disorders have a lower prevalence, as do eating disorders, both affecting 1.2% of the Spanish population. On the other hand, there is insufficient recent national data on other types of mental disorders, such as substance use disorders [5,6].

To address these challenges, effective treatment of mental disorders is crucial, with day hospitals emerging as a key outpatient treatment strategy. Mental Health Day Hospitals are healthcare centers that serve as an intermediate resource between full hospitalization and outpatient treatment, offering a multidisciplinary intervention model that combines the intensity of hospital care with the flexibility of outpatient treatment. This allows patients to receive specialized care through various group and individual therapies while remaining integrated into their social environment, which encourages their involvement and adherence to treatment [7,8]. Some studies have shown that this approach can be effective, finding that participation in day hospital programs can significantly reduce psychiatric symptoms and improve patients′ treatment satisfaction and quality of life compared to traditional outpatient treatment. This approach not only optimizes treatment but also facilitates a more complete and sustainable recovery [9,10,11,12].

These centers have demonstrated the effectiveness of treating a variety of disorders [13], such as mood disorders (e.g., major depression and bipolar disorder), using therapies such as cognitive behavioral therapy and interpersonal therapy to address symptoms and improve emotional regulation [14,15]. Anxiety disorders, including generalized anxiety disorder and panic disorder, are also addressed through graded exposure and stress management techniques. Psychotic disorders, such as schizophrenia, are managed with a combination of antipsychotic medication and psychoeducational and occupational therapy [16,17], while eating disorders, such as anorexia and bulimia, are treated with food-focused cognitive behavioral therapy and family therapy [18,19]. In addition, personality disorders, such as borderline personality disorder, are addressed with dialectical behavioral therapy to develop emotional regulation and interpersonal relationship management skills [20,21,22].

Response to mental health treatment depends not only on the therapeutic intervention, but also on socio-demographic factors that may influence the course and effectiveness of treatment. Previous research has shown that variables such as sex, age, educational level and employment have a significant impact on treatment outcomes [23,24,25]. For example, studies have indicated that women tend to seek treatment more frequently and often report greater symptom severity in depressive and anxiety disorders compared to men [23,26]. Age also plays an important role; older adults often face unique challenges such as comorbidity with physical illness and stigmatization, which can affect the effectiveness of interventions [25,27].

Educational level has been associated with individuals′ ability to access and benefit from treatment, as those with more education tend to have a better understanding of their disorders and greater adherence to treatment recommendations [25].

Finally, employment status also plays a role; those who are employed tend to have greater social support and a daily structure that may facilitate recovery, while unemployment may be associated with a higher risk of mental health deterioration [27].

However, although a few studies have explored this topic in depth, there is a relative scarcity of large-scale, long-term evaluations in European or Spanish contexts. In this context, the objectives of this study are to assess the effectiveness of the Mental Health Day Hospital program in reducing psychiatric symptoms and improving psychological functioning across major diagnostic groups, as well as to examine how patient characteristics influence outcomes. The main hypothesis of the study is that the day hospital is an effective resource for reducing the various symptoms suffered by mental health patients. The relevance of this study lies in its potential to provide empirical evidence to support the effectiveness of day hospitals as a treatment strategy, as well as to offer a deeper understanding of how individual patient characteristics affect treatment outcomes. The findings could contribute to the improvement of mental health intervention programs and also facilitate the personalization of treatments to maximize their impact and improve patients′ quality of life.

## 2. Materials and Methods

### 2.1. Study Design

This was a retrospective, single-center study with repeated cross-sectional assessments with pre- and post-tests, which used the database accumulated up to 2022, corresponding to the last 27 years of the Mental Health Day Hospital of the University Hospital of Burgos. This database was subjected to an exhaustive cleaning process to guarantee the consistency and validity of the information. This process included the digitalization of paper records and a thorough review of clinical histories in order to complete missing data and correct possible inconsistencies. The main objectives of the study were to assess the effectiveness of the Mental Health Day Hospital program in reducing psychiatric symptoms and improving psychological functioning across major diagnostic groups, as well as to examine how patient characteristics influence outcomes.

### 2.2. Sample Size and Participants

The target population of the study comprised all psychiatric patients over 18 years of age referred to the Mental Health Day Hospital of the University Hospital of Burgos, a service that acts as an intermediate step between outpatient consultations and hospitalization, providing intensive and specialized care to patients with various psychiatric pathologies.

No specific sampling was applied, since the aim was to analyze the totality of the data available in the database in order to obtain a comprehensive view of the effectiveness of the service. The only exclusion criterion was the inability to complete the information corresponding to the various assessment tools used, as a result of the severity of their mental disorder. However, the number of patients excluded for this reason was not recorded.

The total sample included 1629 patients registered in the day hospital database who had completed at least all the pre-test information. However, many of these patients did not complete the post-test for various reasons, so follow-up and comparisons between scores were not possible. Complete pre-test and post-test records were collected for only 653 patients. The total sample was used to calculate descriptive data and correlations between variables, while the complete data were used to analyze differences in scores and the effectiveness of the day hospital.

### 2.3. Procedure

Data collection was carried out exhaustively and systematically over the last 27 years, following a specific methodology in the admission and treatment of patients.

Upon receipt of the patient’s referral to the service, an assessment of the patient′s motivation and the urgency of the treatment needed was made, to subsequently schedule an initial appointment for the complete assessment.

At this initial appointment, a comprehensive initial assessment was performed, through a psychiatric, psychological, nutritional and social evaluation, with the patient in some cases with family members, using structured interviews and other standardized assessment tools.

Subsequently, an individualized therapeutic project was developed, through the design of a specific treatment plan for each patient, based on the results of the initial evaluations. A therapeutic contract was then formalized, establishing the treatment objectives and the responsibilities of both the patient and the medical team.

For follow-up and evaluation, daily psychology, psychiatry and social work consultations were held to monitor the evolution of each patient. There was also a final evaluation of the effectiveness of the treatment, through the application of post-treatment psychological tests and adjustments to the therapeutic plan as needed.

### 2.4. Variables and Assessment Tools

Throughout the study, information was collected on several variables, which were measured using the assessment tools commonly used in the Psychiatry Department of University Hospital of Burgos. These assessment tools are as follows:

Sociodemographic information: Sex and age. Each patient was classified as male or female, and age at admission to the service was collected.

Duration of treatment: Measured in days, from admission to discharge from the day hospital.

Main psychiatric diagnosis: The diagnostic criteria were consistent throughout the study. Patients were diagnosed, following the DSM-IV Diagnostic Manual [28], according to the type of psychiatric pathology that caused their admission to the service. The pathologies were classified into mood disorders (major depression, dysthymia, etc.), substance use disorders (substance dependence, alcoholism, etc.), eating disorders (anorexia nervosa, bulimia nervosa, etc.), anxiety disorders (panic disorder, generalized anxiety disorder, etc.) and schizophrenia and other psychotic disorders (schizoaffective disorder, etc.).

Psychological and psychopathological symptoms: Assessed using the Symptom Checklist-90-R (SCL-90-R) [29], a 90-item self-report scale designed to assess a wide range of psychological problems and symptoms of psychopathology. The items are organized into nine primary dimensions: somatization, obsession-compulsion, interpersonal sensitivity, depression, anxiety, hostility, phobic anxiety, paranoid ideation, and psychoticism. Higher scores on each of the dimensions imply a greater number and severity of symptoms.

Anxiety: Assessed by the State-Trait Anxiety Inventory (STAI) [30,31], a 40-item questionnaire that assesses two types of anxiety, state-anxiety (how the patient feels at a given moment), and trait-anxiety (how the patient feels generally). Higher scores mean higher levels of anxiety.

Locus of Control: Assessed by the Locus of Control Scale (LCS) [32], a 29-item questionnaire that measures the difference between an internal locus of control (belief that the patient controls his or her own destiny), and an external locus of control (belief that external factors or fate control the outcome of the patient’s events). Higher scores imply a greater internal locus, while lower scores imply a greater external locus.

Assertiveness: assessed by the Rathus Assertiveness Schedule (RAS) [33], a 30-item scale that assesses patients’ ability to express their feelings, stand up for their rights, and act in a socially appropriate manner without excessive anxiety. Higher scores mean greater assertiveness.

Impulsiveness: Assessed by The Barratt Impulsiveness Scale-11 (BIS-11) [34], a 30-item self-report scale designed to assess different aspects of impulsiveness, including cognitive and motor impulsiveness and lack of planning, as well as total score. Higher scores on the dimensions and on the total scale mean higher impulsiveness.

Self-esteem: Assessed by the Rosenberg Self-Esteem scale (RSE) [35], a 10-item questionnaire that measures the patient’s level of self-esteem. Higher scores mean a higher level of self-esteem.

### 2.5. Statistical Analysis

Statistical analysis was performed using SPSS version 30 (IBM-Inc., Chicago, IL, USA). All quantitative variables were subjected to the Kolmogorov–Smirnov normality test, and most of them followed a non-normal distribution.

First, the main baseline sociodemographic and clinical data were presented. Categorical variables were presented as a number of cases and percentage of the total, while quantitative variables were presented with medians and interquartile ranges. To analyze whether there were baseline differences between the different psychiatric pathology groups, comparisons were made using Chi-square tests and Kruskal–Wallis tests.

To analyze the correlation between the different quantitative variables in the study, Spearman correlations were used. To interpret the correlation indexes, those above ±0.80 were considered very high, those between ±0.60 and ±0.80 were considered high, those between ±0.40 and ±0.60 were considered medium, those between ±0.20 and ±0.40 were considered low, and those below ±0.20 were considered very low.

To analyze intra-group differences and thus be able to verify the evolution of the patients, and consequently the effectiveness of the service intervention, Wilcoxon signed-rank tests were performed. The pre-test scores, those obtained on admission to the service, were compared with the post-test scores, those obtained after completion of the intervention at discharge from the service.

To analyze the inter-group differences, the change score was calculated for each of the variables, finding the differences between the pre-test and post-test. Subsequently, the differences between the different groups of psychiatric pathologies were analyzed using Quade’s non-parametric ANCOVA tests, considering as covariates the initial pre-test scores obtained for each of the variables.

## 3. Results

### 3.1. Baseline Sample Characteristics

Table 1 shows the main sociodemographic and clinical characteristics of the study participants.

As can be seen in the table, more women (56.6%) than men (43.3%) were seen in the day hospital during this period. The mental disorders with the highest female predominance were eating disorders, while those with the highest male predominance were substance use disorders (67.9%).

In terms of age, the group of pathologies whose patients had the highest median age were mood disorders (43 years), while the lowest median age was for eating disorders (23 years).

On the other hand, treatment time in the day hospital was longer for anxiety disorders (108 days), and shorter for substance use disorders (79 days).

### 3.2. Correlations Between Variables

Table 2 shows the correlation between the main quantitative variables.

Age and duration of treatment correlated very poorly with most of the scores obtained in the different assessments performed.

The different symptoms of the SCL-90-R obtained statistically significant correlations, which were high and medium among them, medium with anxiety, medium and low with impulsivity, low with locus of control and with self-esteem, and low and very low with self-esteem.

The two measures of anxiety obtained statistically significant correlations, which were high with each other, low with locus of control, impulsivity and self-esteem, and very low with assertiveness.

Locus control also obtained statistically significant differences with the rest of the variables, which were all low and very low.

Assertiveness did not correlate statistically significantly with impulsivity but correlated poorly with self-esteem.

Finally, the different areas of impulsivity correlated significantly in a medium and low way with each other, and also in a low way with self-esteem.

### 3.3. Intra-Group Differences

Table 3 shows the intra-group differences between the evaluation performed on patients on admission to the unit and the evaluation performed on discharge from the service, thus proving the efficacy of the intervention.

As can be seen in the table, statistically significant differences were obtained in most of the variables (*p* < 0.001). After the intervention, patients in all mental disorder groups reduced all the symptoms of the SCL-90-R assessment, reduced anxiety levels, increased their internal locus, decreased their impulsivity, and increased their self-esteem.

However, hardly any significant results were obtained in the assertiveness assessment. In none of the groups did it increase, except in anxiety disorders, which did (*p* = 0.045).

Finally, statistically significant results were barely obtained in the enhancement of self-esteem. It only improved in the mood disorders group (*p* = 0.021).

### 3.4. Inter-Group Differences

Table 4 shows the inter-group differences for all the change scores obtained in the different assessments, thus verifying which groups of pathologies have improved more or less during the intervention at the Mental Health Day Hospital.

In most of the variables, no statistically significant differences (*p* > 0.05) were found between the groups, specifically in the SCL-90-R assessments of somatization, obsessions/compulsions, interpersonal sensitivity, depression, phobic anxiety, paranoid ideation and psychosis, and also in locus of control, assertiveness, self-esteem, motor impulsiveness, and lack of planning.

Regarding anxiety symptoms of the SCL-90R, patients with schizophrenia and other psychotic disorders improved less than those with mood disorders (*p* = 0.013) and with anxiety disorders (*p* = 0.003).

Regarding the hostility symptoms of the SCL-90R, patients with schizophrenia and other psychotic disorders also improved less than those with mood disorders (*p* = 0.029), with anxiety disorders (*p* = 0.003), and with eating disorders (*p* = 0.005).

As for state anxiety, patients with substance use disorders improved more than those with anxiety disorders (*p* = 0.002), and with schizophrenia and other psychotic disorders (*p* = 0.017). In addition, patients with eating disorders also improved more than those with anxiety disorders (*p* = 0.001), and those with schizophrenia and other psychotic disorders (*p* = 0.011).

Regarding trait anxiety, patients with anxiety disorders improved significantly less than those with mood disorders (*p* = 0.035), eating disorders (*p* < 0.0001), and schizophrenia and other psychotic disorders (*p* = 0.022), while patients with mood disorders also improved less than those with eating disorders (*p* = 0.028).

Finally, regarding cognitive impulsiveness, patients with mood disorders improved less than those with substance use disorders (*p* = 0.007), with anxiety disorders (*p* = 0.002), and with schizophrenia and other psychotic disorders (*p* = 0.002).

## 4. Discussion

Psychiatric disorders currently affect millions of people and represent a difficult challenge for public health worldwide [1]. The main objective of this study was to evaluate the efficacy of the intervention model of the Burgos Mental Health Day Hospital, and to analyze how various sociodemographic factors may influence the evolution and response to treatment.

The results of this study provide an overview of the prevalence of mental disorders according to sex and age, as in other studies [23,24,25]. The attention to a higher number of women compared to men is consistent with the literature, suggesting that certain disorders, especially eating disorders, are more prevalent in women [24]. On the other hand, the high prevalence of substance use disorders in men highlights the need for specific approaches to address sex differences in mental health.

The higher median age observed in patients with mood disorders, in contrast to the lower median in eating disorders, suggests that these disorders may have different developmental trajectories and require interventions tailored to patients’ life stages. This finding may also reflect the chronic nature of mood disorders, which often present later in life.

In terms of treatment duration, anxiety disorders require longer treatment time compared to substance use disorders. This could indicate that anxiety disorders are more complex and require a more intensive therapeutic approach.

Significant correlations between SCL-90-R variables indicate that anxiety, impulsiveness, and self-esteem symptoms are interrelated. However, the low correlation between age and duration of treatment with assessment scores suggests that treatment response may depend more on specific clinical factors than on demographic variables.

As in previous studies, the results obtained show the efficacy of the intervention [9]. The reduction in symptoms in all mental disorder groups is an encouraging finding, although the lack of significant improvement in assertiveness and self-esteem, except in the mood disorder group, suggests that these aspects may require a more specific therapeutic approach.

The inter-group results reveal that, although most variables showed no significant differences between groups, patients with schizophrenia and other psychotic disorders showed less improvement in symptoms of anxiety and hostility compared to other groups. This highlights the need to develop more effective interventions for these patients, who may also benefit from more personalized treatments.

On the other hand, patients with substance use disorders and eating disorders showed significant improvements in anxiety-status, suggesting that the interventions applied may be more effective for these groups. However, patients with anxiety disorders showed less improvement in anxiety-trait, indicating that this group may need a more intensive and targeted approach to address their symptoms.

These findings suggest the need to design personalized therapeutic strategies for psychotic disorders, where improvements were less pronounced, as well as to integrate specific psychosocial modules into mental health intervention programs, such as self-esteem and assertiveness training.

This study has several limitations. First, it is a retrospective, single-center study that analyzes information from the local population only, making it difficult to generalize the results to other populations. Second, the long study period with constantly evolving diagnostic and therapeutic frameworks could cause differences in diagnoses, evaluators, and the methodology used to evaluate the various assessment tools. Third, the inability to follow up and obtain a post-test evaluation in many of the patients evaluated meant that some of the analyses could only be performed with a much smaller sample population. And fourth, this study did not consider the influence of concurrent pharmacological treatments, which could significantly affect the results obtained.

There are several possible directions for future research. On the one hand, it would be appropriate to conduct new prospective, multicenter studies to confirm the findings of this study. On the other hand, it would be appropriate to conduct other types of qualitative studies that explore patients’ perspectives on the care they receive in mental health day hospitals, conducting broader outcome assessments that include recovery, functioning, and quality of life.

## 5. Conclusions

This study highlights the importance of considering differences in sex, age and type of mental disorder when designing interventions in mental health services. The findings could help optimize resources and tailor treatments to maximize therapeutic benefits for patients, thus contributing to improving the quality of psychiatric care in the Mental Health Day Hospital of Burgos. Despite the positive results in symptom reduction, further research is essential to optimize treatments and address the specific needs of each group of patients, especially those with more complex disorders.

## Figures and Tables

**Table 1:** Baseline sociodemographic and clinical characteristics of the sample.

	Mood Disorders N = 377 (23.1%)	Substance Use Disorders N = 349 (21.4%)	Eating Disorders N = 334 (20.5%)	Anxiety Disorders N = 291 (17.9%)	Schizophrenia and Other Psychotic Disorders N = 278 (17.1%)	Total N = 1629 (100%)	Sig. (*p*)
Sex							<0.001
Male, n (%)	125 (33.2%)	237 (67.9%)	25 (7.5%)	138 (47.4%)	182 (65.5%)	707 (43.4%)	
Female, n (%)	252 (66.8%)	112 (32.1%)	309 (92.5%)	153 (52.6%)	96 (34.5%)	922 (56.6%)	
Age; Median (IQ)	43.00 (33.00; 50.00)	41.0 (34.00; 47.00)	23.0 (18.00; 32.00)	38.5 (28.00; 47.25)	30.0 (23.0; 37.25)	35.00 (25.00; 45.00)	<0.001
Duration of treatment; Median (IQ)	107.00 (60.00; 149.50)	79.00 (49.50; 107.00)	105.00 (66.80; 141.50)	108.00 (72.00; 144.00)	102.00 (55.00; 143.00)	98.00 (59.00; 137.00)	<0.001
SCL-90-R_SOM; Median (IQ)	1.58 (0.83; 2.33)	1.16 (0.58; 1.89)	1.41 (0.83; 2.25)	1.75 (1.00; 2.67)	0.83 (0.37; 1.33)	1.33 (0.67; 2.08)	<0.001
SCL-90-R_OBS; Median (IQ)	2.20 (1.50; 2.80)	1.50 (0.90; 2.08)	1.90 (1.20; 2.55)	2.20 (1.50; 2.80)	1.30 (0.70; 2.00)	1.80 (1.10; 2.50)	<0.001
SCL-90-R_IS; Median (IQ)	1.89 (1.11; 2.56)	1.22 (0.77; 2.00)	2.05 (1.33; 2.67)	1.80 (1.22; 2.56)	1.22 (0.58; 2.05)	1.67 (1.00; 2.33)	<0.001
SCL-90-R_DEP; Median (IQ)	2.69 (1.85; 3.23)	1.76 (1.08; 2.46)	2.30 (1.46; 2.92)	2.62 (1.86; 3.23)	1.40 (0.77; 2.23)	2.15 (1.31; 2.92)	<0.001
SCL-90-R_ANX; Median (IQ)	2.00 (1.20; 2.70)	1.45 (0.80; 2.10)	1.70 (0.90; 2.50)	2.00 (1.30; 2.90)	1.00 (0.50; 1.90)	1.60 (0.90; 2.40)	<0.001
SCL-90-R_HOS; Median (IQ)	0.83 (0.50; 2.00)	0.83 (0.50; 1.67)	1.33 (0.66; 2.17)	1.17 (0.50; 2.29)	0.66 (0.17; 1.24)	1.00 (0.50; 2.00)	<0.001
SCL-90-R_PA; Median (IQ)	1.43 (0.57; 2.17)	0.71 (0.28; 1.42)	0.86 (0.29; 1.71)	1.28 (0.57; 2.33)	0.70 (0.20; 1.29)	1.00 (0.42; 1.85)	<0.001
SCL-90-R_PI; Median (IQ)	1.33 (0.83; 2.17)	1.33 (0.70; 2.00)	1.50 (0.83; 1.50)	1.50 (0.83; 2.33)	1.33 (0.50; 2.05)	1.33 (0.81; 2.16)	0.007
SCL-90-R_PSY; Median (IQ)	1.30 (0.80; 2.00)	1.10 (0.60; 1.70)	1.20 (0.70; 1.90)	1.50 (0.80; 2.10)	1.00 (0.40; 1.64)	1.20 (0.70; 1.90)	<0.001
STAI_SAS; Median (IQ)	83.00 (63.00; 94.00)	73.00 (45.00; 89.00)	80.00 (62.00; 93.00	85.00 (65.00; 96.00)	67.00 (35.00; 83.00)	78.00 (55.00; 92.00)	<0.001
STAI_TAS; Median (IQ)	90.00 (70.00; 98.00)	83.00 (62.25; 93.00)	88.00 (70.00; 97.00)	93.00 (75.00; 98.00)	73.00 (40.00; 92.00)	87.00 (62.00; 97.00)	<0.001
LCS; Median (IQ)	12.00 (10.00; 14.00)	11.00 (9.00; 13.00)	12.00 (9.00; 14.00)	12.00 (10.00; 15.00)	11.00 (8.00; 13.00)	12.00 (9.00; 14.00)	<0.001
RAS; Median (IQ)	0.00 (−8.75; 8.00)	3.00 (−5.00; 10.00)	2.00 (−7.00; 10.00)	−1.00 (−10.00; 7.00)	1.00 (−5.75; 8.75)	1.00 (−7.00; 9.00)	<0.001
BIS-11_CI; Median (IQ)	17.00 (14.00; 21.00)	16.00 (13.00; 20.00)	17.00 (13.00; 20.00)	18.00 (14.00; 21.00)	15.00 (12.00; 18.00)	17.00 (13.00; 20.00)	<0.001
BIS-11_MI; Median (IQ)	16.00 (12.00; 22.00)	19.00 (12.50; 24.50)	19.50 (14.00; 26.00)	18.00 (12.00; 24.00)	14.00 (10.00; 20.00)	17.00 (12.00; 23.00)	<0.001
BIS-11_LP; Median (IQ)	18.00 (14.00; 24.00)	20.00 (14.00; 26.00)	19.00 (13.00; 25.00)	19.00 (15.00; 25.00)	20.00 (16.00; 25.25)	19.00 (14.00; 25.00)	0.035
BIS-11_Total; Median (IQ)	52.00 (42.00; 63.00)	56.00 (44.00; 70.00)	56.00 (44.00; 65.00)	54.00 (44.00; 67.00)	51.00 (40.00; 60.00)	54.00 (43.00; 65.00)	0.009
RSE; Median (IQ)	26.00 (21.25; 31.00)	27.00 (24.00; 30.75)	24.00 (20.00; 28.00)	27.00 (21.00; 30.00)	28.00 (24.00; 31.25)	26.00 (22.00; 31.00)	0.002

Abbreviations: IQ = Interquartile Range; SCL-90-R = Symptom Checklist-90-R; SOM = Somatization; OBS = Obsession-compulsion; IS = Interpersonal Sensitivity; DEP = Depression; ANX = Anxiety; HOS = Hostility; PA = Phobic Anxiety; PI = Paranoid Ideation; PSY = Psychoticism; STAI = State-Trait Anxiety Inventory; SAS = State Anxiety Scale; TAS = Trait Anxiety Scale; LCS = Locus of Control Scale; RAS = Rathus Assertiveness Schedule; BIS-11 = Barratt Impulsiveness Scale-11; CI = Cognitive Impulsiveness; MI = Motor Impulsiveness; LP = Lack of Planning; RSE = Rosenberg Self-Esteem scale.

**Table 2:** Spearman correlations between quantitative variables.

	Age	Days Treatment	SCL-90R SOM	SCL-90R OBS	SCL-90R IS	SCL–90R DEP	SCL–90R ANX	SCL–90R HOS	SCL–90R PA	SCL–90R PI	SCL-90R PSY	STAI SAS	STAI TAS	LCS	RAS	BIS-11 CI	BIS-11 MI	BIS-11 LP	BIS-11 Total
Age	−																		
Days treatment	0.006	−																	
SCL-90-R_SOM	0.170 ***	0.080 **	−																
SCL-90-R_OBS	0.109 ***	0.101 ***	0.661 ***	−															
SCL-90-R_IS	−0.047	0.087 ***	0.554 ***	0.735 ***	−														
SCL-90-R_DEP	0.115 ***	0.110 ***	0.666 ***	0.806 ***	0.771 ***	−													
SCL-90-R_ANX	0.110 ***	0.080 **	0.744 ***	0.781 ***	0.720 ***	0.814 ***	−												
SCL-90-R_HOS	−0.095 ***	0.026	0.520 ***	0.563 ***	0.625 ***	0.574 ***	0.614 ***	−											
SCL-90-R_PA	0.091 ***	0.074 **	0.595 ***	0.675 ***	0.678 ***	0.664 ***	0.749 ***	0.482 ***	−										
SCL-90-R_PI	0.002	0.020	0.513 ***	0.620 ***	0.752 ***	0.607 ***	0.652 ***	0.642 ***	0.565 ***	−									
SCL-90-R_PSY	0.105 ***	0.057 *	0.616 ***	0.729 ***	0.744 ***	0.735 ***	0.778 ***	0.607 ***	0.670 ***	0.736 ***	−								
STAI_SAS	0.118 ***	0.074 **	0.456 ***	0.517 ***	0.454 ***	0.576 ***	0.538 ***	0.356 ***	0.416 ***	0.367 ***	0.478 ***	−							
STAI_TAS	0.041	0.069 **	0.389 ***	0.551 ***	0.535 ***	0.601 ***	0.527 ***	0.403 ***	0.431 ***	0.432 ***	0.479 ***	0.664 ***	−						
LCS	−0.028	0.051 *	0.224 ***	0.237 ***	0.256 ***	0.265 ***	0.245 ***	0.199 ***	0.189 ***	0.251 ***	0.233 ***	0.207 ***	0.239 ***	−					
RAS	−0.010	−0.071 **	−0.087 ***	−0.204 ***	−0.253 ***	−0.208 ***	−0.162 ***	−0.019	−0.219 ***	−0.104 ***	−0.172 ***	−0.171 ***	−0.190 ***	−0.130 ***	−				
BIS-11_CI	0.027	0.016	0.360 ***	0.422 ***	0.373 ***	0.394 ***	0.429 ***	0.370 ***	0.351 ***	0.330 ***	0.418 ***	0.265 ***	0.345 ***	0.197 ***	−0.032	−			
BIS-11_MI	−0.021	−0.096 **	0.352 ***	0.327 ***	0.368 ***	0.346 ***	0.426 ***	0.512 ***	0.293 ***	0.418 ***	0.382 ***	0.230 ***	0.304 ***	0.176 ***	0.090 **	0.476 ***	−		
BIS-11_LP	−0.100 ***	−0.068 *	0.158 ***	0.160 ***	0.201 ***	0.148 ***	0.173 ***	0.250 ***	0.137 ***	0.249 ***	0.229 ***	0.095 **	0.158 ***	0.067 *	−0.017	0.339 ***	0.366 ***	−	
BIS-11_Total	−0.048	−0.084 **	0.353 ***	0.361 ***	0.386 ***	0.357 ***	0.409 ***	0.483 ***	0.312 ***	0.415 ***	0.415 ***	0.216 ***	0.312 ***	0.167 ***	0.021	0.679 ***	0.815 ***	0.743 ***	−
RSE	0.028	−0.006	−0.306 ***	−0.389 ***	−0.447 ***	−0.439 ***	−0.366 ***	−0.296 ***	−0.341 ***	−0.334 ***	−0.367 ***	−0.335 ***	−0.371 ***	−0.174 ***	0.227 ***	−0.238 ***	−0.253 ***	−0.150 ***	−0.273 ***

* *p* < 0.05; ** *p* < 0.01; *** *p* < 0.0001. Abbreviations: SCL-90-R = Symptom Checklist-90-R; SOM = Somatization; OBS = Obsession-compulsion; IS = Interpersonal Sensitivity; DEP = Depression; ANX = Anxiety; HOS = Hostility; PA = Phobic Anxiety; PI = Paranoid Ideation; PSY = Psychoticism; STAI = State-Trait Anxiety Inventory; SAS = State Anxiety Scale; TAS = Trait Anxiety Scale; LCS = Locus of Control Scale; RAS = Rathus Assertiveness Schedule; BIS-11 = Barratt Impulsiveness Scale-11; CI = Cognitive Impulsiveness; MI = Motor Impulsiveness; LP = Lack of Planning; RSE = Rosenberg Self-Esteem scale.

**Table 3:** Wilcoxon signed rank tests to analyze intra-group differences.

	Mood Disorders			Substance Use Disorders			Eating Disorders			Anxiety Disorders			Schizophrenia and Other Psychotic Disorders		
	Pre-Test Median	Post-Test Median	Sig. (*p*)	Pre-Test Median	Post-Test Median	Sig. (*p*)	Pre-Test Median	Post-Test Median	Sig. (*p*)	Pre-Test Median	Post-Test Median	Sig. (*p*)	Pre-Test Median	Post-Test Median	Sig. (*p*)
SCL-90-R_SOM	1.58	0.83	<0.001	1.16	0.66	<0.001	1.41	0.75	<0.001	1.75	1.00	<0.001	.83	0.42	<0.001
SCL-90-R_OBS	2.20	1.40	<0.001	1.50	1.00	<0.001	1.90	1.20	<0.001	2.20	1.40	<0.001	1.30	0.80	<0.001
SCL-90-R_IS	1.89	1.22	<0.001	1.22	0.78	<0.001	2.05	1.33	<0.001	1.80	1.33	<0.001	1.22	0.72	<0.001
SCL-90-R_DEP	2.69	1.53	<0.001	1.76	1.08	<0.001	2.30	1.38	<0.001	2.62	1.54	<0.001	1.40	0.77	<0.001
SCL-90-R_ANX	2.00	1.00	<0.001	1.45	0.70	<0.001	1.70	0.80	<0.001	2.00	1.16	<0.001	1.00	0.50	<0.001
SCL-90-R_HOS	0.83	0.33	<0.001	0.83	0.41	<0.001	1.33	0.67	<0.001	1.17	0.67	<0.001	0.66	0.25	<0.001
SCL-90-R_PA	1.43	0.57	<0.001	0.71	0.42	<0.001	0.86	0.29	<0.001	1.28	0.64	<0.001	0.70	0.29	<0.001
SCL-90-R_PI	1.33	0.83	<0.001	1.33	0.83	<0.001	1.50	1.00	<0.001	1.50	1.00	<0.001	1.33	0.67	<0.001
SCL-90-R_PSY	1.30	0.70	<0.001	1.10	0.70	<0.001	1.20	0.70	<0.001	1.50	0.80	<0.001	1.00	0.40	<0.001
STAI_SAS	83.00	55.00	<0.001	73.00	44.00	<0.001	80.00	40.00	<0.001	85.00	63.00	<0.001	67.00	40.00	<0.001
STAI_TAS	90.00	72.00	<0.001	83.00	65.00	<0.001	88.00	60.00	<0.001	93.00	80.00	<0.001	73.00	48.00	<0.001
LCS	12.00	11.00	<0.001	11.00	9.00	<0.001	12.00	10.00	<0.001	12.00	11.00	<0.001	11.00	10.00	0.013
RAS	0.00	3.00	0.094	3.00	4.00	0.194	2.00	3.00	0.615	−1.00	0.50	0.045	1.00	3.00	0.620
BIS-11_CI	17.00	15.00	0.002	16.00	12.50	<0.001	17.00	14.00	0.002	18.00	14.00	<0.001	15.00	13.00	<0.001
BIS-11_MI	16.00	14.00	<0.001	19.00	14.00	<0.001	19.50	15.00	<0.001	18.00	12.00	<0.001	14.00	11.00	<0.001
BIS-11_LP	18.00	16.00	<0.001	20.00	15.50	<0.001	19.00	17.00	0.010	19.00	17.50	0.002	20.00	18.00	<0.001
BIS-11_Total	52.00	44.00	<0.001	56.00	44.00	<0.001	56.00	47.00	<0.001	54.00	45.00	<0.001	51.00	44.00	<0.001
RSE	26.00	29.00	0.021	27.00	29.50	0.063	24.00	23.00	0.414	27.00	28.00	0.127	28.00	29.00	0.648

Abbreviations: SCL-90-R = Symptom Checklist-90-R; SOM = Somatization; OBS = Obsession-compulsion; IS = Interpersonal Sensitivity; DEP = Depression; ANX = Anxiety; HOS = Hostility; PA = Phobic Anxiety; PI = Paranoid Ideation; PSY = Psychoticism; STAI = State-Trait Anxiety Inventory; SAS = State Anxiety Scale; TAS = Trait Anxiety Scale; LCS = Locus of Control Scale; RAS = Rathus Assertiveness Schedule; BIS-11 = Barratt Impulsiveness Scale-11; CI = Cognitive Impulsiveness; MI = Motor Impulsiveness; LP = Lack of Planning; RSE = Rosenberg Self-Esteem scale.

**Table 4:** Quade’s non-parametric ANCOVA tests to analyze inter-group differences.

	Mood Disorders	Substance Use Disorders	Eating Disorders	Anxiety Disorders	Schizophrenia and Other Psychotic Disorders	F	Sig. (*p*)
SCL-90-R_SOM	−0.42	−0.33	−0.50	−0.42	−0.25	2050	0.085
SCL-90-R_OBS	−0.50	−0.30	−0.40	−0.50	−0.40	2205	0.067
SCL-90-R_IS	−0.34	−0.22	−0.44	−0.46	−0.34	2423	0.057
SCL-90-R_DEP	−0.69	−0.46	−0.69	−0.77	−0.46	1225	0.298
SCL-90-R_ANX	−0.50	−0.50	−0.50	−0.70	−0.40	2734	0.028
SCL-90-R_HOS	−0.33	−0.34	−0.50	−0.34	−0.33	2747	0.027
SCL-90-R_PA	−0.42	−0.14	−0.28	−0.29	−0.28	1611	0.169
SCL-90-R_PI	−0.33	−0.33	−0.33	−0.33	−0.50	1728	0.142
SCL-90-R_PSY	−0.40	−0.25	−0.40	−0.40	−0.30	1662	0.157
STAI_SAS	−19.00	−22.00	−20.00	−15.00	−14.50	4091	0.003
STAI_TAS	−11.50	−11.00	−16.00	−7.00	−12.00	3540	0.007
LCS	0.00	−1.00	−1.00	−2.00	−1.00	1995	0.093
RAS	1.00	1.00	0.00	2.00	0.00	0724	0.576
BIS-11_CI	−1.00	−1.00	−1.00	−3.00	−2.00	3693	0.006
BIS-11_MI	−2.00	−3.00	−2.00	−3.00	−2.00	2243	0.063
BIS-11_LP	−2.00	−4.00	−1.00	−1.00	−1.00	2400	0.059
BIS-11_Total	−4.00	−9.00	−5.00	−5.00	−5.00	1539	0.189
RSE	2.00	4.00	2.00	−2.50	0.00	0380	0.822

Abbreviations: SCL-90-R = Symptom Checklist-90-R; SOM = Somatization; OBS = Obsession-compulsion; IS = Interpersonal Sensitivity; DEP = Depression; ANX = Anxiety; HOS = Hostility; PA = Phobic Anxiety; PI = Paranoid Ideation; PSY = Psychoticism; STAI = State-Trait Anxiety Inventory; SAS = State Anxiety Scale; TAS = Trait Anxiety Scale; LCS = Locus of Control Scale; RAS = Rathus Assertiveness Schedule; BIS-11 = Barratt Impulsiveness Scale-11; CI = Cognitive Impulsiveness; MI = Motor Impulsiveness; LP = Lack of Planning; RSE = Rosenberg Self-Esteem scale.

## Data Availability

The complete database for this article is available. Requests for data access should be directed to the corresponding authors (agbustillo@ubu.es) or (jejavier@ubu.es).
